# High-performance ultra-violet phototransistors based on CVT-grown high quality SnS_2_ flakes†

**DOI:** 10.1039/c9na00471h

**Published:** 2019-08-21

**Authors:** Haoting Ying, Xin Li, Yutong Wu, Yi Yao, Junhua Xi, Weitao Su, Chengchao Jin, Minxuan Xu, Zhiwei He, Qi Zhang

**Affiliations:** College of Materials & Environmental Engineering, Hangzhou Dianzi University Xiasha Higher Education Zone Hangzhou 310018 P. R. China lixin@hdu.edu.cn qizhang@hust.edu.cn; College of Science, Hangzhou Dianzi University Xiasha Higher Education Zone Hangzhou 310018 P. R. China

## Abstract

van der Waals layered two-dimensional (2D) metal dichalcogenides, such as SnS_2_, have garnered great interest owing to their new physics in the ultrathin limit, and become potential candidates for the next-generation electronics and/or optoelectronics fields. Herein, we report high-performance UV photodetectors established on high quality SnS_2_ flakes and address the relatively lower photodetection capability of the thinner flakes *via* a compatible gate-controlling strategy. SnS_2_ flakes with different thicknesses were mechanically exfoliated from CVT-grown high-quality 2H-SnS_2_ single crystals. The photodetectors fabricated using SnS_2_ flakes reveal a desired response performance (*R*_*λ*_ ≈ 112 A W^−1^, EQE ≈ 3.7 × 10^4^%, and D* ≈ 1.18 × 10^11^ Jones) under UV light with a very low power density (0.2 mW cm^−2^ @ 365 nm). Specifically, SnS_2_ flakes present a positive thickness-dependent photodetection behavior caused by the enhanced light absorption capacity of thicker samples. Fortunately, the responsivity of thin SnS_2_ flakes (*e.g.* ∼15 nm) could be indeed enhanced to ∼140 A W^−1^ under a gate bias of +20 V, reaching the performance level of thicker samples without gate bias (*e.g.* ∼144 A W^−1^ for a ∼60 nm flake). Our results offer an efficient way to choose 2D crystals with controllable thicknesses as optimal candidates for desirable optoelectronic devices.

## Abstract 2

High-performance UV photodetectors established on CVT-grown SnS_2_ flakes provide an efficient way to choose 2D crystals with an optimal thickness.

## Graphical abstract

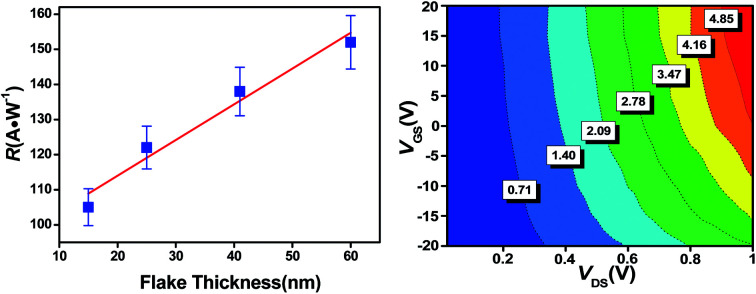

## Introduction

Two-dimensional (2D) materials, such as graphene since its first isolation from bulk crystals, have created opportunities to access novel features at reduced dimensionality.^1–3^ Inspired by this, there has been growing interest to find new 2D structures, because fundamentally and technologically interesting properties (charge carriers and photons are confined in a 2D plane, *etc.*) have been identified in the ultrathin limit, but most attention so far has been paid to layered transition metal dichalcogenides (TMDs).^4–7^ Furthermore, graphene shows a zero-band gap which traditionally has limited its application in optoelectronic devices.^8^ In addition, layered semiconducting TMDs with a general description of MX_2_ (a metal atom, M = Mo, W, Sn, *etc.* is sandwiched between two adjacent chalcogen layers X = S, S, Te, *etc.*) have exhibited extraordinary properties in many fields, emerging as suitable candidates for next-generation electronic or photoelectronic systems beyond the current silicon era.^9–13^

The surprising and appealing characteristics found in the low-dimensional limit stimulate the exploration of other van der Waals layered materials.^14–16^ Nowadays, various layered semiconductors can be prepared *via* top-down (*e.g.* mechanical exfoliation) or bottom-up (*e.g.* chemical vapour deposition, CVD) methods.^17,18^ Nevertheless, in view of sustainable development, earth-abundant 2D materials are more essential for widespread use in modern devices. Tin disulfide (SnS_2_, belonging to the IV–VIA group) comprises earth-abundant constituents (Sn and S), which could vastly support the industrial requirements.^19^ Bulk SnS_2_ crystals (usually representing a bandgap of 2.1–2.31 eV) have long been explored for possible applications in photovoltaics and photoelectrochemistry.^20,21^ In addition, recent evidence has already pointed out the fascinating progress in applying 2D SnS_2_ structures in lithium ion batteries,^22^ field-effect devices^23^ and photodetectors,^24^ benefitting from the simple exfoliation from bulk crystals and the controlled bottom-up synthesis.^25,26^ In particular, due to a considerable absorption coefficient (10^6^ cm^−1^),^27^ light sensors using 2D SnS_2_ structures (most are obtained by CVD) exhibit superior UV-vis sensing performance such as high responsivity and good stability.^28–30^ Note that, however, the thickness dependent responsivity of 2D material (including SnS_2_ flakes) based photodetectors (*i.e.* the relatively lower photodetection capability in thinner flakes) may make them inappropriate for the upcoming smart systems with high integration density because the chip manufacturing process in development requires thinner (monolayer of few-layer) semiconductor channels.^31–33^ Therefore, some prospective strategies should be proposed to address this problem and make these prototypes appealing for practical applications. Unfortunately, this issue has not been well considered in previous optoelectronic applications.

In this paper, we have achieved high-performance UV photodetectors established on SnS_2_ flakes and employed a gate-tunable strategy to address the challenge posed by the positive thickness-dependent light sensing behaviour of 2D materials. SnS_2_ flakes with different thicknesses (that can be thinned to ∼7 nm in the work) were mechanically exfoliated from CVT-grown high-quality 2H-SnS_2_ single crystals. We describe several characterization methods to identify the phase and microstructure of these multilayer SnS_2_ samples. The photodetectors fabricated by using individual SnS_2_ flakes reveal a desired response performance (*R*_*λ*_ ≈ 112 A W^−1^, EQE ≈ 3.7 × 10^4^%, and D* ≈ 1.18 × 10^11^ Jones) under UV light with a very low power density (0.2 mW cm^−2^ @ 365 nm). Specifically, the SnS_2_ flakes present a relatively low photodetection ability in the thinner flakes. But it is found that the responsivity of thin flakes (*e.g.* ∼15 nm) could be enhanced to ∼140 A W^−1^ aided by a positively gated bias (+20 V), and it is comparable to the performance of a non-gated ∼60 nm flake. This indeed provides a potential way to address the positive thickness-dependent detection capability mainly caused by the enhanced light absorption capacity of thicker samples, and our findings imply that such earth-abundant and environmentally friendly tin-based chalcogenides are desirable for sustainable “green” optoelectronics applications.

## Experimental section

### Preparation of multilayer SnS_2_ flakes

The starting materials here are SnS_2_ bulk crystals, grown *via* a chemical vapor transport (CVT) route with pure iodine as the transport agent. The pre-mixed powders of Sn (99.99%, Aladdin) and S (99.99%, Alfa Aesar) at a stoichiometric ratio of 1 : 2 with additional iodine (99.8%, Aladdin, 5 mg cm^−3^) were vacuum sealed (>10^−4^ Torr) in a quartz tube. The quartz tube was then placed in a two-zone furnace. The reactant zone was slowly heated up (∼10 h) to 800 °C while the other end was set to 750 °C. The growth process was maintained for ∼10 h, followed by a naturally cooling process down to room temperature. Afterwards, strip-like products were obtained. Thin SnS_2_ flakes were mechanically exfoliated from the as-synthesized crystals aided by adhesive tape and then dry-transferred onto a freshly cleaned SiO_2_/Si substrate (with a dielectric layer, ∼300 nm thick SiO_2_). Thin samples were roughly identified using an optical microscope (Olympus, CX41) in combination with a charge-coupled device.

### Materials characterization

The phases of the crystals were characterized by X-ray diffraction (XRD) on a Rigaku Miniflex 600x powder diffractometer. XPS measurements were performed using a Thermo ESCALAB 250XI X-ray photoelectron spectrometer. The thicknesses of the exfoliated SnS_2_ samples were measured by atomic force microscopy (AFM, Agilent 5500). A home-built Raman spectroscope/microscope (iHR320, Horiba) was utilized to acquire Raman spectra and spatially resolved Raman maps with an incident laser of 532 nm while employing a 405 nm light for photoluminescence (PL) measurements. A transmission electron microscope (TEM, JEM-2100F) was employed to evaluate the morphologies and crystal structure of thin SnS_2_ flakes. The UV-vis spectrum was measured using a spectrophotometer (MPC-3100, Shimadzu).

### Device fabrication and measurements

The fabrication of photodetectors based on individual SnS_2_ flakes relies on a standard UV lithography (URE-2000/25) process followed by thermal evaporation (JSD 300) of desired electrode metals (Cr/Au, ∼10/60 nm). The photoresponse measurements were executed on a probe station (ZFT-50T) equipped with two sourcemeters (Model 2450, Keithley). Light sources (THORLABS) of different wavelengths with tunable power were applied in the photodetection tests.

## Results and discussion

**Fig. 1:**
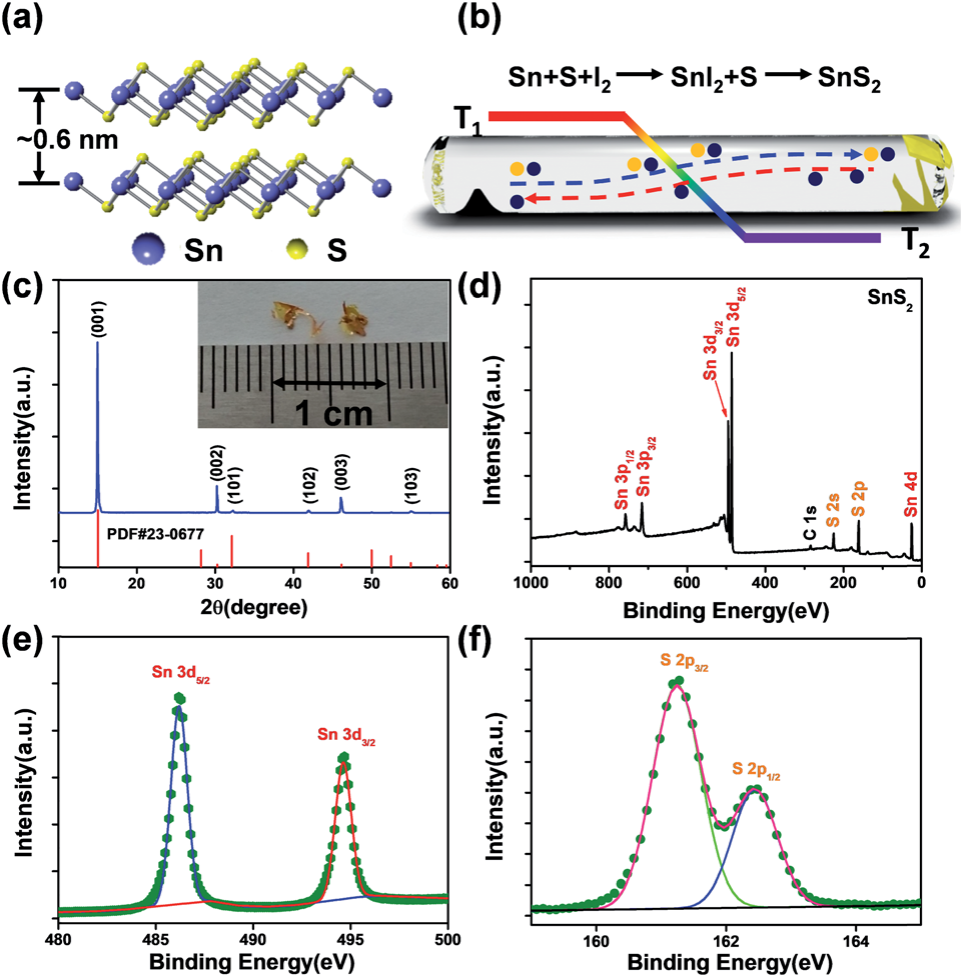
Synthesis of SnS_2_ crystals. (a) Crystal structure (side view) of layered SnS_2_. (b) Schematic diagram showing the CVT process. (c) XRD patterns of the as-synthesized products, inset: the photograph of SnS_2_ single crystals. (d) XPS survey of SnS_2_ crystals, (e) Sn3d XPS and (f) high resolution S 2p XPS of SnS_2_ single crystals.

To get SnS_2_ samples with varied thicknesses, we employed tape-assisted mechanical isolation from raw crystals synthesized *via* a chemical vapour transport (CVT) approach. Previous research suggests that SnS_2_ occurs as different polytypes associated with different interlayer stacking of S–Sn–S layers; a low-temperature (<800 °C) synthesis process tends to produce the 2H-polytype.^34,35^Fig. 1a illustrates the 3D structure model of 2H-SnS_2_; the S–Sn–S atomic planes (the distance between adjacent planes is ∼0.6 nm) with covalent bonding are held together by weak van der Waals force.^36^ In this work, SnS_2_ crystals were prepared *via* a CVT process in a two-zone tube furnace, as schematically shown in Fig. 1b (more information has been provided in the Experimental section). During the CVT growth, gas transport is realized by temperature gradient and thus impact on the final products (Fig. S1†). In this work, a 50 °C temperature gradient is in favour of growing high quality SnS_2_ single crystals, in agreement with a previous report,^37^ and large sized crystals (given in the inset of Fig. 1c, lateral size > 3 mm) with a clean surface could be found at the lower temperate zone. The phase structure of the crystals was identified through the powder X-ray diffraction (XRD) technology, as depicted in Fig. 1c. The products exhibit a hexagonal structure (JCPDS PDF number 23-0677, *a* = *b* = 0.36 nm, *c* = 0.59 nm). Strong diffraction reflections emerging around ∼15.0°, ∼30.3° and ∼46.1° are indexed to the (0001), (0002) and (0003) planes, respectively. The predominance of the (000*N*) (*N* = 0, 1, 2, *etc.*) peaks suggests that the *Z*-direction with the (0001) plane as the basal plane is the preferential orientation for these growing SnS_2_ crystals.^38^ The valence states of the SnS_2_ crystals were characterized by X-ray photoelectron spectroscopy (XPS). The spectra have been calibrated by employing absorbed C (1s) as the reference. Expected Sn and S elements from these crystals were detected in the XPS survey (Fig. 1d). Two peaks at 486.5 and 495.1 eV (Fig. 1e) originate from Sn3d_5/2_ and Sn3d_3/2_ of Sn^4+^.^39^Fig. 1f illustrates the high-resolution core XPS spectrum of S 2p (∼162.6 eV), an indication of the existence of S2p_3/2_ and S2p_1/2_ orbitals.^40^ These results evidence the formation of layered SnS_2_ crystals with high quality.

**Fig. 2:**
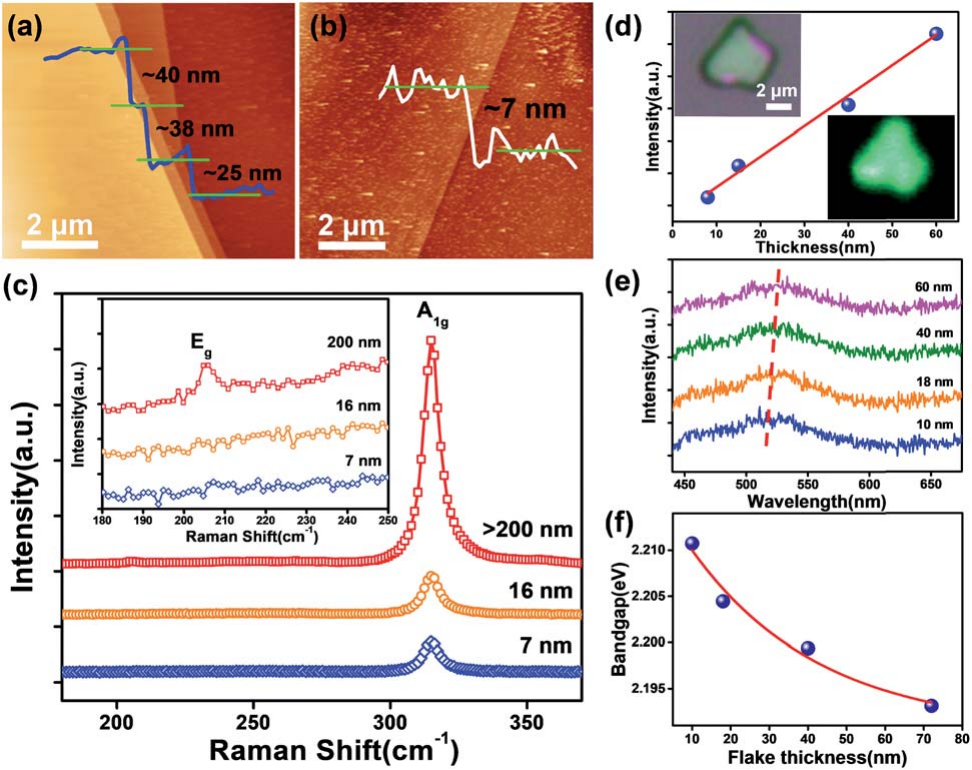
Characterization of SnS_2_ flakes. (a and b) Typical AFM images of ultrathin SnS_2_ flakes acquired *via* mechanical exfoliation. (c) Thickness dependent Raman spectra of SnS_2_ flakes, inset: enlarged view of the characteristic peak at ∼205 cm^−1^. (d) Change in the intensity of the A_1g_ peak with layer thickness, inset: an optical image of a SnS_2_ flake and the corresponding Raman map of the A_1g_ mode. (e) PL spectrum of SnS_2_ with different thicknesses. (f) The layer-dependent bandgap of few-layer SnS_2_.

Thin flakes with varied thicknesses (Fig. 2a) were obtained through mechanical exfoliation from large crystals exceeding 10 μm (limited by the AFM scanning range) accompanied by a clean surface and pristine state. The height of multilayer SnS_2_ flakes often presents lateral dimension changes shown in Fig. 2b measured at the edge between the thicker and thinner part indicating a thickness of ∼7 nm (corresponding to about ten layers). Raman spectroscopy was employed to quantify and map SnS_2_ flakes. Previous reports suggest the existence of different crystal polytypes for layered SnS_2_ crystals, 4H- and 2H-phases, respectively.^41^ The most intense Raman peak for the 4H polytype emerges at 313.5 cm^−1^, relative to the joint contribution of A_1_ and E phonon modes (this peak is very close to the A_1g_ mode of 2H-SnS_2_ at 315 cm^−1^), while the E-mode generates a doublet at 200 and 214 cm^−1^, respectively.^19^ In parallel, in 2H-SnS_2_ crystals, the E_g_ mode generates a single, intense line around 205 cm^−1^, allowing a facile distinction against the 4H polytype.^28^ The observed Raman active modes in Fig. 2c, *i.e.* two main peaks at 205 (E_g_, in plane) and 315 cm^−1^ (A_1g_, out of plane) occur in the thick flakes (>200 nm, the inset of Fig. 2c), hence, providing an unambiguous fingerprinting of 2H polytypic crystals in this work. Here, the E_g_ modes weaken and become unobservable with the reduction of flake thickness (down to the nanometer level), which could be presumably attributed to the reduction of in-plane scattering centers in the ultrathin SnS_2_ flakes.^42,43^ But the A_1g_ (out of plane) mode illustrates a significant increase in the peak intensity with the increasing thickness (Fig. 2d), probably arising from the enhanced light absorption capacity of the thicker samples,^44,45^ which may have an influence on the device performance. Raman mapping (Fig. 2c) of a SnS_2_ flake (the optical image is depicted in Fig. 2d) using the characteristic line at 315 cm^−1^ demonstrates its uniform polytype. 2H-SnS_2_ flakes exhibit isotropy in the (0001) plane as confirmed by the polarized Raman characterization of the A_1g_ vibration mode. Therefore, the origination dependent photo-responsivity should not be a problem for their application in photodetection.^46^ The PL spectra of 2D SnS_2_ flakes in Fig. 2e consist of a single feature, attributed to the nature of their indirect band-gap structure, with a value of ∼2.20 eV similar to the reported data of 2H-SnS_2_.^28^ Moreover, the peak shifts to lower energies with increasing thickness (Fig. 2f). During PL measurements, holes will combine with the photo-excited electrons *via* Coulomb interactions with the binding energy lying in the band-gap region; however, strong spatial confinement and a reduced screening effect in ultrathin samples could result in a significantly enhanced excitonic effect causing a blue shift in the bandgap.^47^

**Fig. 3:**
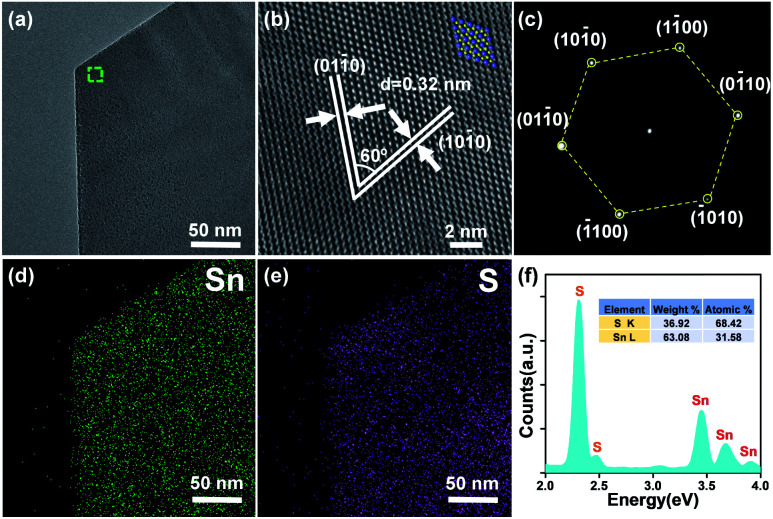
TEM identification of SnS_2_ flakes. (a) Low-magnification TEM image of an ultrathin SnS_2_ flake. (b) High resolution TEM characterization with atomic mode on the top and (c) the corresponding SAED pattern of the SnS_2_ flake. EDX elemental mapping of (d) Sn and (e) S revealing uniform distributions. (f) EDX spectrum of the SnS_2_ flake; the inset illustrates the atomic ratio of Sn and S.

The microscopic structure and the compositions of the 2D SnS_2_ crystals were determined using a transmission electron microscope (TEM) and an energy-dispersive X-ray detector (EDX). Fig. 3a shows a low magnification view (bright field) of a single flake with no observable terraces. A direct view of the lattice structure (Fig. 3b, reflected by the HRTEM image of the labeled region in Fig. 3a) suggests the hexagonal arrangement (atomic mode given on the top) of Sn and S atoms in 2H SnS_2_ crystals. The plane distance of ∼0.32 nm well matches the *d*-spacing of the {10 − 10} planes for hexagonal phase SnS_2_.^48^ The selected area electron diffraction (SAED) pattern (obtained by applying incident electrons parallel to the *c*-axis) in Fig. 3c shows well-sequenced diffraction spots with a six-fold symmetry, indicating high-quality crystallinity of this crystal caused by a vertically stacking layer plane along the [0001] direction. The composition of the crystals was verified by adopting energy-dispersive X-ray spectroscopy (EDX). EDX mapping results in Fig. 3d and e suggest the homogeneous distribution of Sn and S elements in the flake. In parallel, the EDX spectrum (Fig. 3f) clearly reveals signals of Sn and S with an approximate atomic ratio of 2 : 1. The aforementioned demonstrations signify the high purity of the single-crystalline SnS_2_ flakes.

**Fig. 4:**
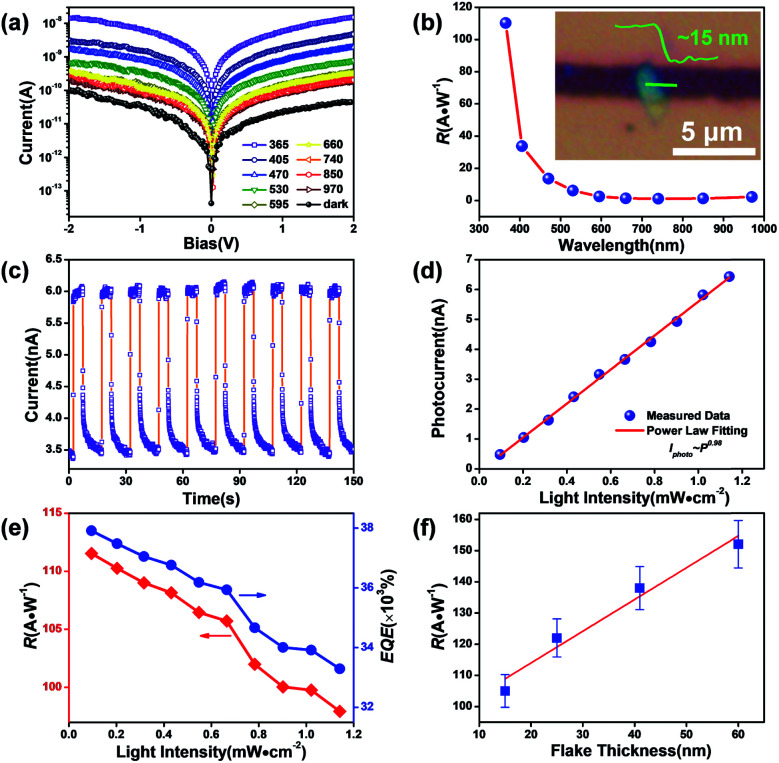
Photodetectors established on ultrathin SnS_2_ flakes. (a) *I*–*V* characteristics measured in darkness and under incident light of varied wavelengths (under comparable light intensity). (b) Spectral responsivity of the SnS_2_ flake based photodetector, inset: the representative two-terminal devices designed on a thin SnS_2_ flake. (c) Time resolved current of the light sensor measured at *V*_DS_ = 1 V under 365 nm (0.2 mW cm^−2^). (d) Power law fitting photocurrents *versus* light intensities. (e) Responsivity (*R*_*λ*_) and external quantum efficiency (EQE) plotted as a function of light intensity. (f) Thickness dependent responsivity of SnS_2_ flake based devices.

Photodetectors established on individual flakes were employed to systematically estimate the optoelectronic properties of the SnS_2_ crystals. The set-up diagram of a two-terminal light sensor is illustrated in Fig. S3a,† where the incident light is perpendicular to the SnS_2_ flake. Fig. S3b† and 4a present the current–voltage (*I*_DS_–*V*_GS_) characteristics of the representative SnS_2_ light sensor in darkness and under illumination with varied wavelengths. The linear curves result from the near ohmic barrier between the Cr/Au electrodes and SnS_2_ channel. The plot of responsivity (Fig. 4b, *R*_*λ*_ = *I*_ph_/*PS*, *I*_ph_ = *I*_light_ − *I*_dark_, *S* refers to the activated area of ∼5.17 μm^2^ and *P* is the power intensity of incident light, *V*_DS_ = 1 V) *versus* wavelength provides a quantitative assessment of the photoresponse ability of the SnS_2_ flake. The optical image of the SnS_2_ channel with a thickness of ∼15 nm and a length of ∼3 μm is illustrated in the inset. The cut-off wavelength is about 550 nm, close to its deduced bandgap, *E*_indirect_ ≈ 2.18 eV from the UV-vis absorption spectrum in Fig. S4.†^29^ The light sensor exhibits high photo responsivity in the ultra-violet range. The stability and reproducibility of the SnS_2_ based photodetector (Fig. 4c, measured at a bias of 1 V) are uncovered *via* tracking current under periodic illumination of 0.2 mW cm^−2^ @ 365 nm. Enlarged views of the rising and decay sides are illustrated in Fig. S3c,† from which the response time (*τ*_rising_) and recovery time (*τ*_decay_) are calculated to be 40 and 160 ms, respectively. Fig. 4d displays the dependence of photocurrent on light intensity, which can be well described by a power law, *I*_ph_ ∼ *P*^*β*^, where *β* is an exponent determined by trap states on the surface of the photo-sensitive media.^49^ In general, a low power intensity would benefit the occupation of surface states by holes separated from photo-induced electron–hole pairs, followed by a rapid recombination with the negatively charged oxygen. But abundant electron–hole pairs will be generated under a higher light intensity. The reduction of the hole-trap states at the surface until the complete occupation of surface traps will contribute to faster recombination (in several picoseconds) between extra charges; subsequently, these fresh pairs will not contribute to the charge transfer process, but form non-radiative carrier-recombination centers, thus leading to a decline (Fig. 4e) of responsivity and quantum efficiency (EQE = *hcR*_*λ*_/*eλ*, *h* is Planck's constant).^50,51^ The sub-linear behaviour in Fig. 4d with a fitting value *β* ∼ 0.98 (very close to 1) may be associated with a low concentration of traps or defects in these SnS_2_ flakes.^52^ The parameters of our sensors are comparable or superior to those of documented 2D SnS_2_ and other 2D material-based UV sensors as summarized in Table 1, potentially offered by the high-quality, large specific surface area.^53^ In addition, we find a positive thickness-dependent photodetection capability of these 2D SnS_2_ flakes with a similar active zone (*R*_*λ*_ up to 144 A W^−1^ for a ∼60 nm thick SnS_2_ flake, in Fig. 4f), which has also been documented in other research.^54^ For a given wavelength range, the photocurrent of photosensitive flakes is proportional to the absorption if we consider a constant namely the internal quantum efficiency (*η*), which can be expressed as *I*_ph_ = *α* × *d* × *η*, where *α* is the absorption and *d* is the thickness of the flake.^55^ Therefore, the dependence of photoresponsivity on flake thickness may originate from enhanced absorption in thicker samples.^56^

**Table 1:** Comparison of the 2D UV photodetector performance with those reported by others. *T*: thickness, ME: mechanical exfoliation, D*: detectivity

Material	Synthesis	Electrode	*T* [nm]	*λ* [nm]	*R*_*λ*_ [A W^−1^]	EQE [%]	D* [Jones]	*τ* [ms]	Ref.
WO_3_	CVD	Cr/Au	12	365	293	997	—	40/80	57
β-Ga_2_O_3_	Oxidant	Cr/Au	6	254	3.3	1.6 × 10^3^	4 × 10^12^	30/60	58
Bi_2_Te_3_	ME	Pt	—	325	26.82	102	1.29 × 10^9^	280/1600	59
SnS_2_	CVD	Ti/Au	—	390	—	150	—	8/150	60
SnS_2_	CVD	Cr/Au	10	350	260	9.3 × 10^4^	1.9 × 10^10^	20/16	28
SnS_2_	CVD	Cr/Au	114	100–800	1.568	480.1	—	42/40	61
SnS_2_	ME	Cr/Au	15	365	112	3.7 × 10^4^	1.18 × 10^11^	40/160	This work

**Fig. 5:**
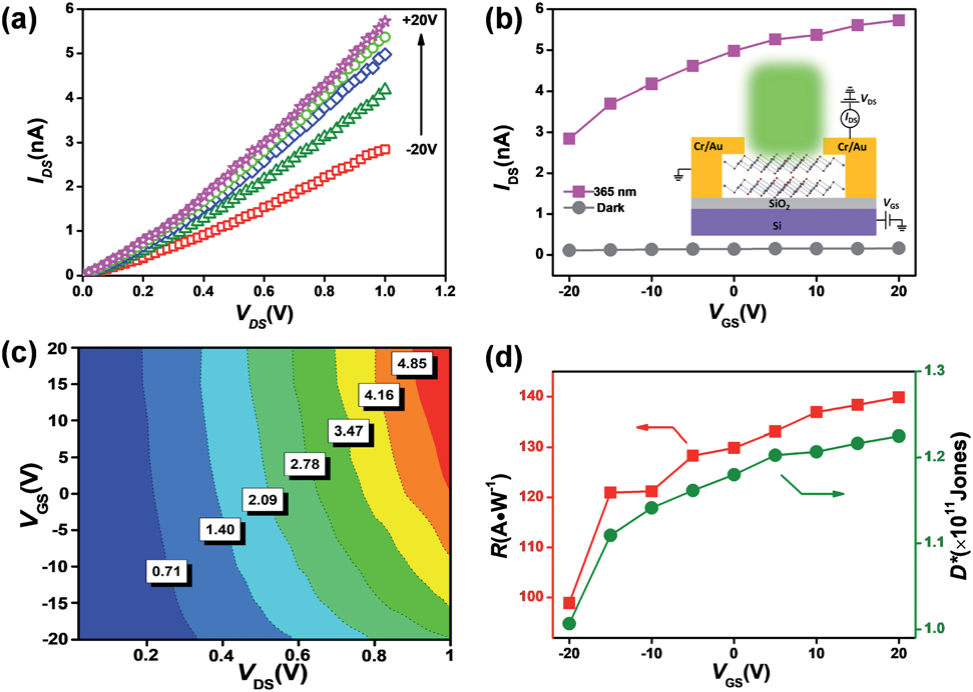
Phototransistors based on SnS_2_ flakes. (a) Output characteristics of the SnS_2_ (∼15 nm) transistors operated under incident light of 0.2 mW cm^−2^ @ 365 nm. (b) *I*_DS_–*V*_GS_ curves under 365 nm light and in darkness at *V*_DS_ = 1 V, inset: the layout of the phototransistor. (c) False-color plot of the SnS_2_ phototransistor exposed to incident light, where the color reflects the intensity of the measured photocurrent. (d) Photoresponsivity and detectivity measured at *V*_DS_ = 1 V as a function of *V*_GS_.

The positive thickness-dependent photodetection capability may be a challenge for the system-on-a-chip design where a thinner channel is required. Previous work employed the surface sensitization of SnS_2_ nanosheets using a 2 nm thick HfO_2_ nanolayer grown by atomic layer deposition (ALD).^54^ However, this inevitably leads to a complex procedure or a higher production cost. A phototransistor, in which the increase of gate voltage gives rise to an increase in the channel current, may pave an alternative way to address this issue.^62,63^ Here, the emphasis has been placed on the device (as schematically shown in the inset of Fig. 5b) performance operating under illumination (0.2 mW cm^−2^ @ 365 nm). The light output characteristics (thickness ∼ 15 nm) are displayed in Fig. 5a. The output and transfer characteristics measured in darkness are illustrated in Fig. S5.† Both the SnS_2_ channels reveal a typical n-type semiconducting behaviour. The correlation between the channel current and back-gate bias (*I*_DS_–*V*_GS_ curves) under illumination and dark conditions is plotted in Fig. 5b. An increase in the channel current can be found as *V*_GS_ increases, possibly due to the leading role of photo-generation in comparison with tunneling or thermionic currents.^64^ The false-color plot in Fig. 5c provides direct evidence that higher photocurrent could be achieved under a high (positive) gate bias. Thereby, the indicators of light sensors relying on SnS_2_ flakes, such as responsivity and detectivity (Fig. 5d), could be further improved (up to 140 A W^−1^) through changing the gate voltage. Under a positive gate bias, the Fermi level in the (n-type) semiconductor will approach the conduction band and lead to a reduction of barrier height; as a result, more photo-excited charges could overcome the gate barrier and contribute to an increased carrier density.^65,66^ Hence, this compatible manufacturing approach could address the positive thickness-dependent sensing capability caused by the enhanced absorption capacity in thicker samples.

## Conclusions

In summary, we report high-performance UV photodetectors established on SnS_2_ flakes and address the relatively low photodetection capability in the thinner flakes *via* a compatible gate-tunable route. Multilayer SnS_2_ flakes (that can be thinned to ∼7 nm in this work) were mechanically isolated from CVT-grown high-quality 2H-SnS_2_ single crystals whose components are both inexpensive and earth-abundant. The phase and microstructure were unambiguously identified through several characterization techniques, including XRD, XPS, AFM, Raman and TEM. When exposed to UV illumination with a very low power density (0.2 mW cm^−2^ @ 365 nm), the light sensors using SnS_2_ flakes exhibit high responsivity (112 A W^−1^), EQE (3.7 × 10^4^%) and detectivity (1.18 × 10^11^ Jones), comparable or superior to those of reported SnS_2_ and other 2D material-based UV photodetectors. Most importantly, SnS_2_ flakes present a positive thickness-dependent photodetection behaviour, possibly attributed to the enhanced light absorption capacity of thicker samples. However, the responsivity of thinner flakes (*e.g.* ∼15 nm) can be further improved (up to 140 A W^−1^) under a gate bias of +20 V, comparable with the performance of a non-gated thick flake (∼144 A W^−1^ for a ∼60 nm flake). Hence, our results offer an efficient way to choose 2D materials with an optimal thickness, and such earth-abundant and environmentally friendly tin-based chalcogenides are highly desirable for sustainable “green” optoelectronics applications.

## Conflicts of interest

There are no conflicts to declare.

## Supplementary Material

NA-001-C9NA00471H-s001
