# Supplementary material for: High-performance ultra-violet phototransistors based on CVT-grown high quality SnS2 flakes
Source: Nanoscale Adv. 2019 Aug 21;1(10):3973–9. doi: 10.1039/c9na00471h (PMC9418408; doi:10.1039/c9na00471h)
Supplement: NA-001-C9NA00471H-s001 [file NA-001-C9NA00471H-s001.pdf]

Electronic Supplementary Material (ESI) for Nanoscale Advances.
This journal is © The Royal Society of Chemistry 2019

Supporting Information

High-Performance Ultra-Violet Phototransistors Based on CVT-Grown High Quality SnS_2 Flakes

Haoting Ying, Xin Li, Yutong Wu, Yi Yao, Junhua Xi, Weitao Su, Chengchao Jin, Minxuan Xu, Zhiwei He, Qi Zhang,

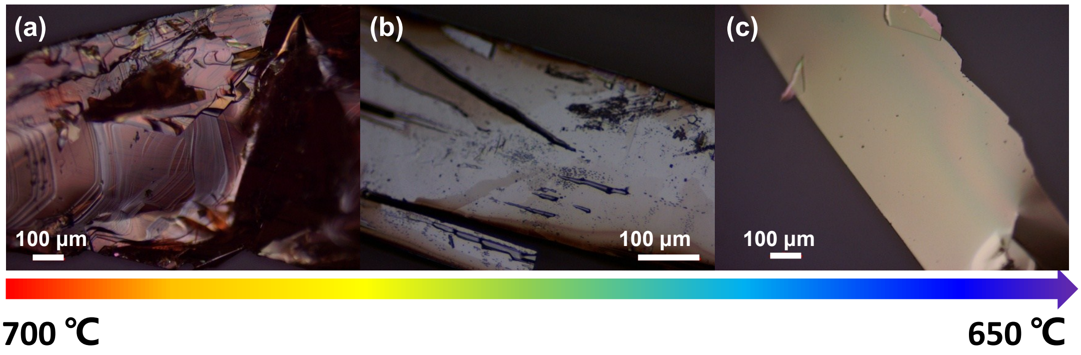

Figure S1. The temperature gradient effect on the final products

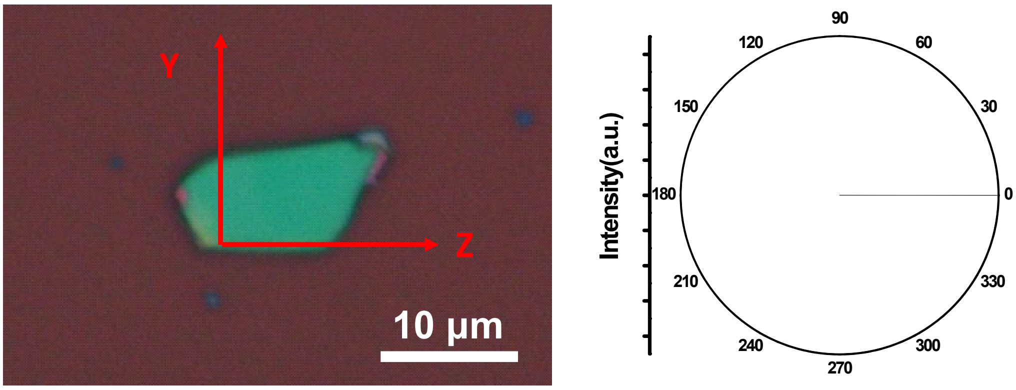

Figure S2. Polarized Raman characterization of A_{1g} vibration mode.

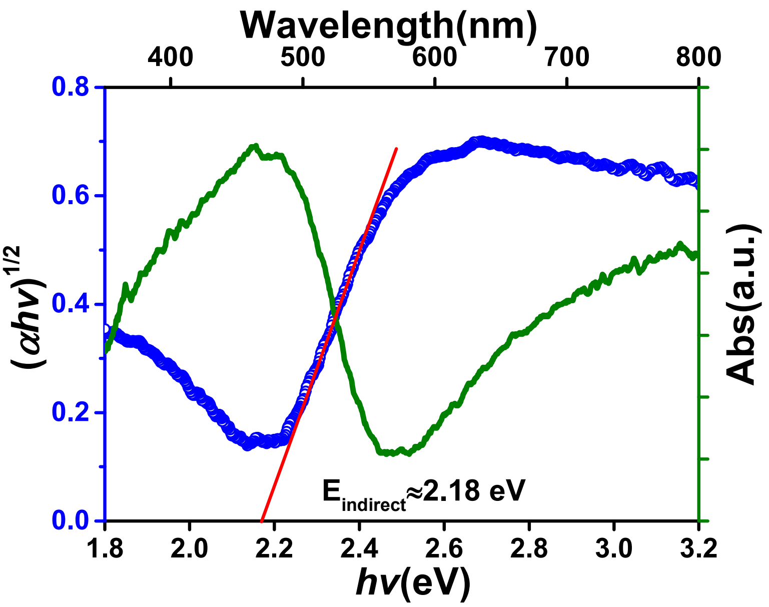

Figure S3. UV–vis absorption spectrum of SnS_2 flakes located on SiO_2/Si substrates with a Kubelka−Munk (K−M) transformation to the bandgap.

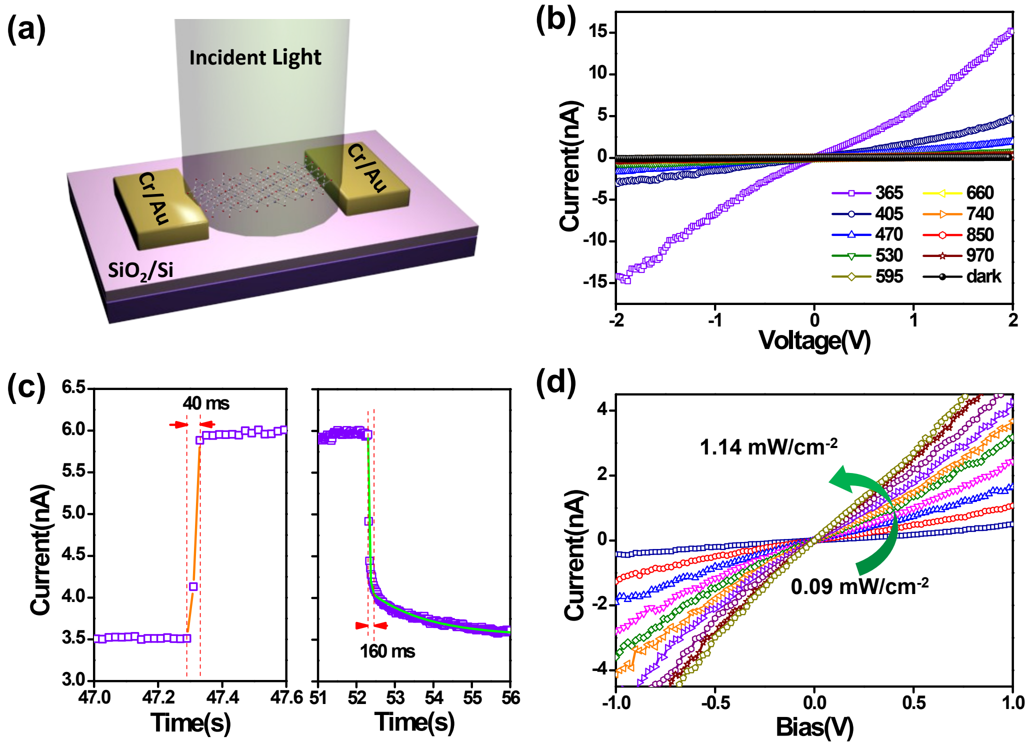

Figure S4. a) The layout of the two-terminal photodetectors based on individual SnS_2 flakes. b) The current versus voltage (I-V) plots of the photodetector in darkness and under incident light of different wavelengths. c) Enlarged views of the rise and decay sides from Figure 4c. d) Light intensity dependent I-V characteristics.

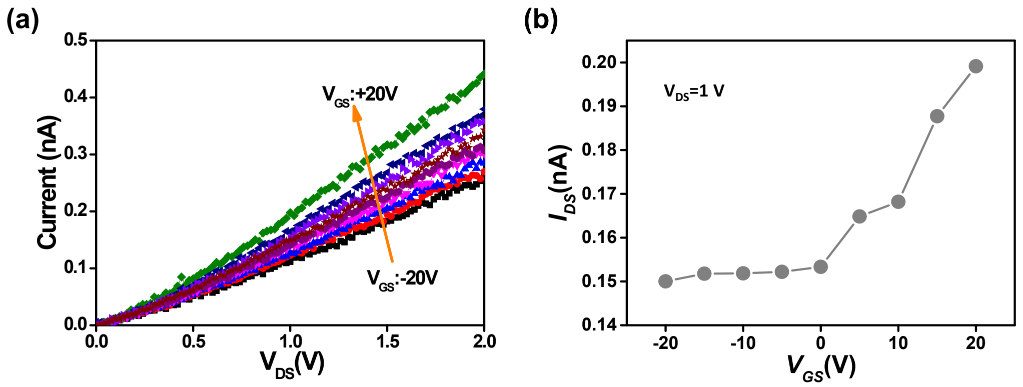

Figure S5. The (a) output and (b) transfer characteristics of SnS_2 flake based transistor measured in darkness.
